# Sublingual house dust mite immunotherapy has no impact on decrease of circulating erythrocytes upon airway allergen challenge in allergic rhinitis

**DOI:** 10.1038/s41598-017-02321-y

**Published:** 2017-05-31

**Authors:** Galateja Jordakieva, Michael Kundi, Patrick Lemell, René Zieglmayer, Petra Zieglmayer, Jasminka Godnic-Cvar, Erika Jensen-Jarolim

**Affiliations:** 1 Department of Physical Medicine, Rehabilitation and Occupational Medicine, Medical University of Vienna, Vienna, Austria; 2 Institute of Pathophysiology and Allergy Research, Center of Pathophysiology, Infectiology and Immunology, Medical University of Vienna, Vienna, Austria; 3 Center for Public Health, Medical University of Vienna, Vienna, Austria; 4 Vienna Challenge Chamber (VCC), Vienna, Austria; 5 The Interuniversity Messerli Research Institute, Medical University Vienna, University of Veterinary Medicine Vienna, University of Vienna, Vienna, Austria

## Abstract

House dust mite (HDM) allergy is a predominant cause for perennial allergic rhinitis (AR) in Europe. We recently reported that circulating erythrocyte numbers decrease after airway allergen challenge in a murine asthma model and in grass-pollen sensitized AR subjects. Consequently, we aimed to evaluate these findings in HDM sensitized AR subjects and the influence of preceding allergen immunotherapy. Seventy-seven (age 26.8 ± 7.3 years; 54.5% female) HDM-allergic rhinitis subjects previously enrolled in a randomized, monocentric sublingual immunotherapy (SLIT) trial at the Vienna Challenge Chamber (VCC) were included. Subjects had either received placebo (n = 22), low-dose HDM (n = 29) or high-dose HDM specific sublingual immunotherapy (n = 26) daily for 24 weeks. Blood sampling was performed before and after 6 hours of HDM allergen exposure. Overall, specific airway allergen challenge resulted in a significant decrease in circulating erythrocytes and hematocrit (p < 0.001), and elevation of leukocytes (p < 0.001), particularly segmented neutrophils (p < 0.001). Gender had no significant effect on the observed changes in circulating blood cells. Erythrocytes decreased and neutrophil counts increased significantly after airway allergen challenge regardless of preceding immunotherapy. These findings imply a rapid systemic mobilization of neutrophils occurring within immediate type hypersensitivity response upon a specific allergen challenge, which is possibly inversely linked with the erythrocyte numbers.

## Introduction

Allergic rhinitis (AR) is the most common cause for chronic rhinitis worldwide, affecting up to 30% of the human population^1, 2^. Most cases of perennial or “non-seasonal” AR in Europe are induced by house dust mite (HDM) allergens and result in a poorer health-related quality of life than seasonal rhinitis in affected subjects^3^. From a pathophysiological perspective, HDM-associated AR is an immunoglobulin E (IgE) mediated upper airway mucosal reaction to structural proteins and fecal proteases predominantly from the species *Dermatophagoides pteronyssinus* (*Der p*) and *Dermatophagoides farinae* (*Der f*) in Europe^3, 4^. Diagnosis of HDM allergen sensitization comprises skin-prick testing and specific IgE analysis on extracts and allergen molecules^5^, the latter overcoming non-specific reactions in SPT^6^. The current therapeutic standards for HDM associated AR include reduction of exposure and the use of immune modulating drugs. While the efficacy of specific allergen immunotherapy and, even more, sublingual immunotherapy (SLIT) were previously disputed^7^, a change of paradigms has taken place with the increasing number of controlled clinical trials conducted in HDM allergy patients using various formulations of HDM SLIT, such as tablets or drops. The clinical efficacy and long-term effects of HDM SLIT in AR is today undisputable in adults in European^8, 9^ and US^10^ studies, as well as in elderly patients^11^ and in children^12–15^.

In the absence of laboratory biomarkers correlating with the clinical success of allergen immunotherapy, clinical parameters, i.e. objective symptom-medication scores, subjective visual analogue scores, and percutaneous, conjunctival and respiratory allergen challenges are the current state of the art methods to determine the effects of allergen immunotherapy. Of those, allergen provocations in allergen challenge chambers represent the gold standard^16–18^. An environmental allergen challenge chamber was recently used to prove the clinical efficacy of a HDM sublingual tablet^15^. After 6-hour exposure the total nasal symptom score was a primary endpoint, the total ocular symptom scores and total symptom scores secondary end points in this study. Samples of this clinical trial were examined in the present study.

Erythrocytes, also known as red blood cells (RBC), represent the most abundant blood cells and are mainly responsible for oxygen-binding and transportation throughout the organism. An effect on, or involvement of, erythrocytes in the immune response after allergen challenge has not yet been investigated in allergic rhinitis, even though the crosstalk between erythrocytes and immune cells has gained interest in the pathophysiology of various immune-mediated diseases^19, 20^. For example, several damage-associated molecular patterns (DAMPs) have been identified in erythrocytes, such as heme, Hsp70 and IL-33, which can be released into the circulating blood upon intravascular hemolysis and potentiate inflammatory responses if not neutralized by specialized scavenger proteins^20^. We have previously reported that numbers of circulating erythrocytes significantly decreased after airway challenge with the specific allergen grass pollen^21^. Our findings deduced from a mouse model and verified in human AR subjects, were primarily attributed to a potential recruitment of erythrocytes to the respiratory mucosa as a site of hypoxia during specific allergen challenge and successive microepistaxis^21^. In accordance, increased free hemoglobin was previously found in nasal lavage samples after allergen challenge in AR subjects as a possible result of increased vascular permeability^22^. In this work we also observed in the human AR subjects that the 4 hour challenge with grass pollen allergens resulted in significant elevation of neutrophils in the peripheral blood, while eosinophil counts remained unchanged.

Consequently we hypothesized that successful allergen immunotherapy could neutralize the effects of an allergen challenge on erythrocytes and neutrophils, and possibly correlate with clinical improvement. The aim of the present study was therefore to i) confirm the previously observed changes in blood cell counts with a different allergen, HDM, in an environmental exposure chamber, and ii) to compare laboratory parameters for RBC and granulocytes before and after HDM challenge in AR subjects being treated with SLIT versus placebo^15^.

## Results

### Subject characteristics

A total of seventy seven (n = 77) Caucasian subjects (54.5% female) with an average age of 26.8 ( ± 7.3) years were evaluated; all had confirmed AR to HDM allergens. According to the preceding drug therapy, subjects were divided in three groups^15^: group 1 had received placebo (n = 22), group 2 low dose (6 developmental units [DU] of MK-8237) (n = 29), and group 3 high dose (12 DU of MK-8237) (n = 26) HDM specific sublingual immunotherapy. Except for a higher proportion of females in the low dose subgroup, subject characteristics, preceding drug therapy and time since drug therapy were similar across groups (Table 1). To determine the lasting clinical benefit, allergen challenges were done in all three subject groups over 6 hours in the Vienna challenge chamber; blood cell counts before (baseline, “pre”) and immediately after the challenges (6 h time point, “post”) were compared. Compared to nasal symptom scores before participation in the sublingual immunotherapy trial, only subjects in group 3 (high dose SLIT) reported significantly less symptoms (−39%) during airway allergen challenge. (Supp. Fig. 3a). No significant differences in nasal symptom scores were found between subjects in group 1 (placebo) and group 2 (low dose SLIT) during airway challenge. (Supp. Fig. 3b+c).

**Table 1:** Characteristics of subjects undergoing airway allergen challenge with house dust mite extract at the Vienna Challenge Chamber (VCC) after participating in a sublingual immunotherapy (SLIT) trial.

	*group 1*	*group 2*	*group 3*	Total
	n = 22	n = 29	n = 26	n = 77
Age	26.2 ± 6.4	26.3 ± 5.7	27.8 ± 9.5	26.8 ± 7.3
Female (%)	8 (36.4%)	22 (75.9%)	12 (46.2%)	42 (54.5%)
Drug in SLIT trial	placebo	6 DU MK-8237	12 DU MK-8237	
Days since SLIT trial	265.04 (94, 488)	248.13 (94, 418)	257.77 (94, 435)	256.22 (94, 488)

Values present mean ± SD or mean (minimum, maximum); *DU* developmental units.

### Allergen challenge and erythrocytes

**Figure 1:**
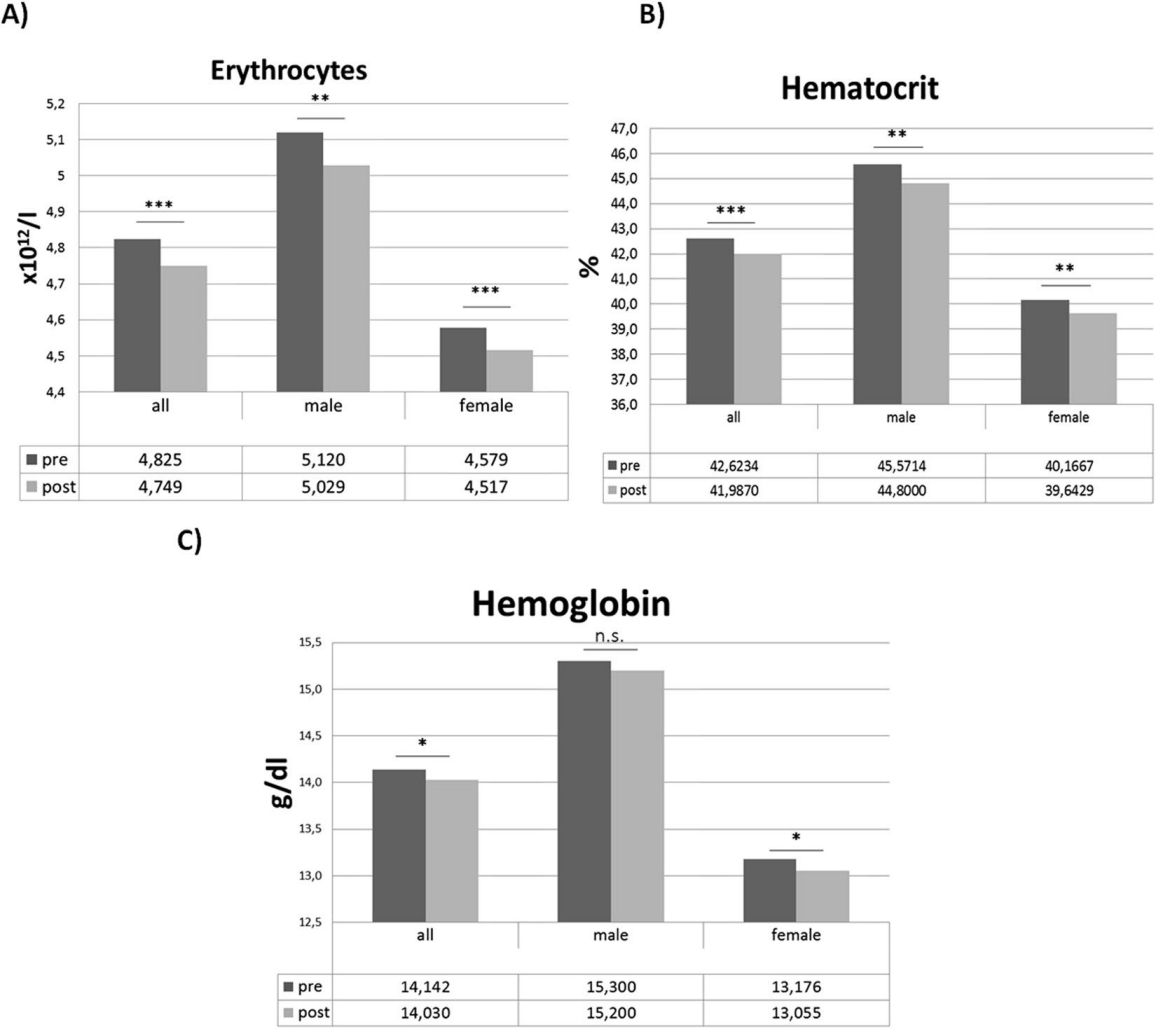
Circulating erythrocytes (**A**), hematocrit (**B**) and hemoglobin (**C**) levels before (pre) and after (post) airway allergen challenge with house dust mite extract in all subjects, as well as in male and female subjects. *p < 0.05; **p < 0.01; ***p < 0.001; n.s. not significant.

Absolute erythrocyte counts, hematocrit (p < 0.001, respectively) and hemoglobin (p = 0.014) values significantly decreased during allergen challenge in all AR subjects, independent of preceding treatment. MCH (mean corpuscular hemoglobin) and MCHC (mean corpuscular hemoglobin concentration) values (p = 0.002, p = 0.006, respectively) declined upon allergen challenge, whereas MCV (mean corpuscular volume) values remained almost constant. The decline in erythrocytes and hematocrit values after allergen challenge was observed similarly in female (p < 0.001 and p < 0.01) and in male (p < 0.01, respectively) subjects (Fig. 1).

In the subgroup analysis, erythrocytes, hematocrit and MCH (p < 0.05), but not hemoglobin (p = 0.081), decreased significantly after allergen challenge in the placebo-treated group.

These changes were likewise observed in the allergen immunotherapy groups: in those treated with low-dose SLIT, a significant decline in erythrocyte counts, hematocrit (p < 0.01, respectively) and hemoglobin (p < 0.05) was found similar to those treated with high-dose SLIT who had significantly lower erythrocyte counts and MCH levels (p < 0.05) after allergen challenge while the hematocrit did not reach statistical significance (p = 0.052).

Comparing the groups, significantly higher baseline levels of erythrocyte and hemoglobin levels were found in male subjects of the placebo group compared to male subjects treated with high dose SLIT (p < 0.05); a similar tendency in female subjects was found but did not reach statistical significance.

### Allergen challenge and leukocytes

Overall, leukocytes and absolute numbers of segmented neutrophils (p < 0.001, respectively) were significantly increased after airway allergen challenge compared to baseline values before challenge. A significant increase of leukocyte and absolute segmented neutrophil numbers after challenge was observed in all groups (p < 0.01, respectively) regardless of preceding placebo or SLIT treatment. Absolute circulating eosinophil numbers slightly decreased (p < 0.05) compared to baseline values only in the placebo group, while in both SLIT treated groups eosinophil counts did not show statistically significant differences after 6 h of allergen challenge (Table 2).

**Table 2:** Circulating blood cell numbers before (pre) and after (post) airway allergen challenge with house dust mite extract.

	placebo			low dose SLIT			high dose SLIT			Total		
	group 1			group 2			group 3					
	n = 22			n = 29			n = 26			n = 77		
	pre	post	*p*	pre	post	*p*	pre	post	*p*	pre	post	*p*
Erythrocytes *x10* ^*12*^ */l*	5.06 ± 0.46	4.96 ± 0.46	*	4.66 ± 0.48	4.58 ± 0.47	**	4,80 ± 0,29	4,76 ± 0,31	*	4.83 ± 0.44	4.75 ± 0.44	***
Hemoglobin *g/dL*	14.78 ± 1.30	14.60 ± 1.22	*n.s*.	13.60 ± 1.35	13.45 ± 1.44	*	14,20 ± 1,04	14,20 ± 1,12	*n.s*.	14.14 ± 1.32	14.03 ± 1.35	*
Hematocrit	0.44 ± 0.03	0.43 ± 0.03	*	0.41 ± 0.04	0.40 ± 0.04	**	0,43 ± 0,03	0,43 ± 0,03	*n.s*.	0.43 ± 0.04	0.42 ± 0.04	***
MCV fl/cell	87.59 ± 5.54	87.86 ± 5.14	*n.s*.	88.52 ± 6.05	88.45 ± 6.46	*n.s*.	89,58 ± 3,62	89,50 ± 3,89	*n.s*.	88.61 ± 5.19	88.64 ± 5.30	*n.s*.
MCH pg/cell	29.23 ± 2.11	29.59 ± 2.36	*	29.34 ± 2.04	29.38 ± 1.99	*n.s*.	29,73 ± 1,69	30,04 ± 1,64	*	29.44 ± 1.94	29.66 ± 1.99	**
MCHC g/dl	33.41 ± 1.00	33.68 ± 1.62	*n.s*.	33.07 ± 0.80	33.34 ± 0.81	*n.s*.	33,04 ± 0,92	33,31 ± 0,97	*n.s*.	33.16 ± 0.90	33.43 ± 1.14	**
Leukocytes *x10* ^*9*^ */l*	6.15 ± 1.28	7.40 ± 1.89	**	6.55 ± 1,80	7.59 ± 1.84	**	5,93 ± 1,49	7,25 ± 1,78	***	6.23 ± 1.57	7.42 ± 1.82	***
*Absolute Counts (x10* ^*9*^ */l)*												
*Segmented*	3.13 ± 0.98	4.46 ± 1.75	***	3.71 ± 1.59	4.51 ± 1.52	**	3,32 ± 1,07	4,48 ± 1,52	***	3.41 ± 1.28	4.49 ± 1.57	***
*Eosinophils*	0.29 ± 0.30	0.25 ± 0.30	*	0.27 ± 0.21	0.26 ± 0.18	*n.s*.	0,21 ± 0,10	0,20 ± 0,11	*n.s*.	0.26 ± 0.21	0.24 ± 0.20	*n.s*.
*Basophils*	0.05 ± 0.03	0.04 ± 0.02	*n.s*.	0.05 ± 0.02	0.04 ± 0.02	*n.s*.	0,05 ± 0,03	0,05 ± 0,03	*n.s*.	0.05 ± 0.03	0.04 ± 0.02	*n.s*.
*Lymphocytes*	2.22 ± 0.55	2.17 ± 0.42	*n.s*.	2.05 ± 0.69	2.22 ± 0.71	*n.s*.	1,91 ± 0,48	2,03 ± 0,48	*n.s*.	2.05 ± 0.59	2.14 ± 0.56	*n.s*.

Values present mean ± SD. *p < 0.05; **p < 0.01; ***p < 0.001; *n.s*. not significant;

*SLIT* sublingual immunotherapy, *MCV* mean corpuscular volume, *MCH* mean corpuscular hemoglobin, *MCHC* mean corpuscular hemoglobin concentration.

## Discussion

We previously reported a decrease in circulating erythrocytes after prolonged airway challenge with a specific allergen in grass pollen sensitized AR patients and, in analogy, in a murine model^21^. Our present study in HDM sensitized AR subjects confirms this phenomenon for an independent allergen. Moreover, the observed effect on red blood cells was not influenced by preceding allergen-specific immunotherapy compared to treatment with placebo. Therefore, the decline in red blood cells is not suitable as a biomarker for allergen immunotherapy, but is rather a surrogate for mucosal hyper-reactivity upon contact with respiratory allergens in sensitized subjects. Moreover, although total erythrocyte counts and hemoglobin levels in the peripheral blood were lower at the end of allergen challenge compared to baseline values before challenge, mean values remained in the normal reference ranges for the general population. To our knowledge, this is the first study in AR subjects sensitized to HDM reporting significant changes in circulating red blood parameters after HDM allergen challenge. These findings are consistent with the results of our previous study in grass-pollen sensitized AR subjects after allergen challenge, who had not undergone allergen specific immunotherap^21^. Both our studies together imply that the effect of allergen challenge on circulating erythrocyte levels is not limited to either HDM or grass pollen allergen, but rather represents involvement of RBC in a general pathophysiological mechanism.

A short-term decline of circulating erythrocytes after allergen exposure as found in our study can have several explanations. In acute allergic rhinitis mast cell induced inflammatory mediators, such as leukotrienes and prostaglandin, cause vascular hyper-permeability, vasodilation and chemotaxis of neutrophils and eosinophils to the local inflammation site following allergen exposure^23^. The increased microvascular leakage primarily leads to plasma extravasation making an acute systemic increase in hematocrit values more likely than a decrease, as found in our study. Thus, a dilution effect is unlikely. Further, an effect on erythropoiesis can be ruled out due to the relatively short duration between the two time points of blood sampling (6 hours of allergen challenge). In our setting, a removal of erythrocytes from the circulating blood stream seems the most likely explanation for the observed periphery decrease. Cysteinyl-leukotrienes (CysLTs), lipid inflammatory mediators which are released from immune cells in AR^24, 25^, have been shown to mediate erythrocyte clearance via erythrocyte apoptosis. Eryptosis has previously been described in inflammation and sepsis^26, 27^, but so far not in allergic disease. Foller *et al*. found that CysLTs can activate ion-channels on the erythrocyte surface resulting in Ca^2+^ influx which further leads to cell shrinkage and membrane scrambling^28^. This mechanism could explain an induced clearance of circulating erythrocytes by splenic macrophages and thus decrease in peripheral RBC counts. There were significantly less symptoms in the high dose HDM SLIT group and, following our hypothesis, less CysLTs should lead to less RBC degradation. Indeed, compared to baseline values there was a more prominent mean absolute RBC decline in the placebo (0.10 × 10^12/l^) than in the high dose SLIT group (0.04 × 10^12/l^) after allergen challenge. Furthermore, a subclinical bronchial reaction to airway allergen challenge, resulting in hypoxia, cannot be excluded. In this setting, a pulmonary redistribution of erythrocytes for forced oxygen loading is also a possible scenario.

Interestingly, we also observed changes in MCH and MCHC with inconsistent statistical significance in the subgroup analysis. Since MCH and MCHC are calculated laboratory values dependent on hemoglobin concentration and erythrocyte count (MCH) or hematocrit (MCHC), we attributed these findings to the prominent decline of erythrocyte counts and hematocrit as against more stable hemoglobin levels.

Allergen challenge further resulted in significant elevation of circulating leukocyte counts, specifically the absolute segmented neutrophil numbers in all groups including placebo. An increase in circulating neutrophils after allergen challenge in HDM-associated AR has been described before^29, 30^. As mentioned above, neutrophil chemotaxis to the site of infection or allergen-associated inflammation is an early innate immune response to potential pathogens, resulting in the induction of inflammatory cells and cytokines responsible for oxidative stress at the inflammation site^29, 30^. Further, the release of chromatin-based extracellular traps from neutrophils (NETs) as a defense mechanism against pathogens has recently gained interest in various diseases. In inflammatory respiratory diseases, NETs have been shown to form in the capillary plexuses of the lungs^31^ and can potentially entangle with red blood cells^32^. Dismantling NETs by DNase treatment improved lung function in murine asthma models and in some *in vivo* human studies^31^. A trapping of erythrocytes in neutrophil derived NETs at the site of inflammation, i.e. in the capillary plexuses of the nasal mucosa, could potentially explain a fraction of the decrease in their circulating counts. This trapping could further result in local hemolysis and release of DAMPs from erythrocytes^20^ potentially influencing the inflammatory reaction. A potential association between neutrophil recruitment and circulating erythrocyte decrease in specific allergen challenge would need to be evaluated in further studies.

Although subjects who had undergone high-dose immunotherapy (group 3) reported less rhinitis symptoms during allergen challenge, (Fig. S3) the decrease of circulating erythrocytes was statistically unaffected by preceding SLIT. Interestingly, however, there was a significant difference in erythrocyte and hemoglobin levels between the placebo and the high-dose SLIT group at baseline, with higher levels in placebo group AR. Probably due to higher subject numbers in both groups, this finding only reached statistical significance in male subjects. Elevation of red blood cells and oxygen-binding hemoglobin is a well-known mechanism for compensation of chronic low oxygen supply. Chronic hypoxic conditions resulting from living in high altitude areas, chronic obstructive pulmonary disease, but also cigarette smoking, have since long been associated with secondary erythrocytosis^33^. In allergic asthma, alterations in oxidant/antioxidant balance have been shown to shift towards increased oxidative stress, with increased superoxide dismutase activity and decreased glutathione peroxidase activity in erythrocytes^34^. Chronic allergic rhinitis partially limits the nasal airflow, but cannot result in relevant hypoxia if the respiratory airflow is unaffected. Accordingly, in our study, mean erythrocyte, hematocrit and hemoglobin levels remained in the normal reference ranges at both sampling times in all groups. However, the significantly higher baseline levels for all three parameters in untreated (placebo) compared to symptom reduced (SLIT) AR subjects, might indicate a subclinical difference in oxygen supply due to availability of additional nasal airflow. Another explanation for this observation might be a direct effect of the immunological therapy on the red blood cells in the SLIT groups, but no significant differences were found between red blood parameters in the high-dose and the low-dose SLIT groups, and surprisingly, the lowest mean values of erythrocyte counts, hemoglobin and hematocrit were found in the low dose SLIT group; altogether, the hypothesis of a dose-dependent drug effect is not support by the data.

In our study, there were no significant differences between female and male subjects apart from the known sex-dependent physiological divergence in erythrocyte counts, hemoglobin and hematocrit levels. While similar trends were found in all treatment subgroups, statistical significance was not always reached after further subdivision into male and female subjects.

Finally, only the eosinophil counts correlated with the treatment status of the subjects. Upon allergen challenge circulating eosinophil numbers remained unchanged in both HDM SLIT groups but slightly decreased in the placebo group. Eosinophil recruitment from the blood circulation and their arrest on activated endothelium and extravasation has been described in allergic airway diseases^23, 35^. There are various explanations for the mechanisms of allergen immunotherapy. T_regs_^36^ and B_regs_^37^ as sources of immunomodulatory cytokines IL-10 and TGFb counterregulate the Th2 cytokine dominance in allergy, including the Th2 cytokine IL-5. IL-5 is an important activator for eosinophils and acts in the nasal mucosa, especially in the late allergic reaction^38^. As was recently demonstrated in a mouse model^39^, SLIT could contribute to an overall lower Th2 bias.

## Conclusion

To the best of our knowledge this is the first study addressing peripheral blood cell counts following allergen challenge in AR subjects after specific immunotherapy. Overall our study shows that (i) circulating erythrocyte numbers significantly decrease after specific allergen challenge in allergic rhinitis subjects, possibly due to leukotriene induced eryptosis; (ii) leukocyte counts, particularly segmented neutrophils, increase after allergen challenge, independent of preceding immunotherapy; (iii) placebo-treated subjects – possibly compensatory - have higher baseline levels of erythrocytes than subjects after effective SLIT treatment, (iv) only eosinophil dynamics differed between subjects after immunotherapy and subjects who had received placebo. These observations are in accordance with our previous findings on RBC dynamics after allergen challenge and imply an involvement of erythrocytes and neutrophils in the acute allergic response.

## Methods

### Subjects

In this monocentric study, otherwise healthy human subjects (n = 77) with a medical history of HDM allergy were recruited at the Vienna Challenge Chamber (VCC)^15^.

HDM allergen sensitization (Der p, Der f, or both) was assessed by specific IgE assessment and skin prick testing prior to study inclusion. All evaluated subjects had previously participated in a randomized, double-blind pharmacological trial on dose-related efficacy of HDM sublingual immunotherapy tablets^15^ and had received either placebo, low dose (6 DU) or high dose (12 DU) HDM sublingual immunotherapy (MK-8237 [Merck/ALK-Abelló]) for a total of 24 weeks. Data presented in the current study were assessed in volunteering subjects 3 to 16 months (⦰ 8.4 months) after MK-8237 trial completion (24 weeks SLIT and additional 2-weeks follow-up). (Table 1) The HDM allergen challenges were approved by the ethical committee “Österreichische Arbeitsgemeinschaft für Klinische Pharmakologie” (1811/2013) and data collection was conducted between 11/2013 and 10/2014 with volunteer subjects after gaining written informed consent of each participant. All studies in the VCC are strictly conducted in conformance with GCP (Good Clinical Practice).

#### Inclusion criteria

- age 18–65 years
- healthy individuals, except for allergic rhinitis and intermittent or mild asthma not requiring treatment and associated with normal baseline lung function (FEV1 ≥ 70% of predicted)
- positive skin prick test (wheal ≥ 3 mm compared to standard control [saline solution]) and specific IgE ( ≥ 0.7 kU/l, radioallergosorbent test class [RAST] ≥ 2) results for *Der p* and/or *Der f* within the last 12 months preceding the study

#### Exclusion criteria

- nasal abnormalities (septum perforation, polyps, malformations)
- cold symptoms or acute infections of the upper respiratory tract within 3 weeks
- active respiratory tract disease, other than mild asthma not requiring treatment
- ongoing immunotherapy and/or immunomodulating medication

### Interventions

#### Airway Allergen Challenge

A mixture of equal parts of *Der p* and *Der f* whole bodies (Allergon, Thermo Fisher Scientific, Ängelholm, Sweden) and a smaller part of dermatophagoides feces extract (Citeq Biologics, Groningen, The Netherlands) (ratio 10:10:1) was used for allergen provocation in the challenge chamber. Allergen products used for the mixture were provided by their respective manufacturers including certificates of analysis and dry stored at +2 to +10 °C (whole bodies) or frozen at −25 °C (feces) in their original containers. Airway allergen challenge was conducted in the VCC (a 54 m^3^ sealed provocation chamber) for 6 consecutive hours as previously described. (21) Airborne HDM allergen mixture concentration was monitored and kept constant. Every 15 minutes allergic symptoms, i.e. total nasal symptom score (TNSS - sneezing, obstructed, runny and itchy nose) were evaluated. Further, every 30 minutes nasal air flow volume (active anterior rhinomanometry) and every 60 min bronchial air flow volume (spirometry) values were assessed for safety reasons.

#### Blood Cell Collection

Blood was drawn immediately before and after 6 h of allergen challenge from periphery veins and sampled in ethylenediamine tetraacetic acid (EDTA) coated tubes. EDTA blood samples were shortly stored at +2 °C to +10 °C and sent to an ISO 9001-certified laboratory (Labors.at, Vienna, Austria) for differential blood cell count analysis, and determination of hemoglobin [Hb] and hematocrit [Hc].

#### Statistical Analysis

Data were analysed by ANOVA (sigma restricted parameterization) with three factors: group (placebo, low and high dose) and gender as between-subject factors and pre/post challenge as within-subject factor. Comparisons against placebo were performed applying linear contrasts. Normality of residuals was tested by Kolmogorov-Smirnov tests with Lilliefors correction. Homogeneity of variances was tested by Levene’s tests. For relative white blood-cell counts an arcsine transformation was applied. For all statistical tests p < 0.05 was considered significant. The software packages SPSS (version 20.0 for Windows, IBM Corp., USA) and STATISTICA (version 12.0; StatSoft Inc., USA) were used for statistical calculations.

#### Data availability

The datasets generated during and analysed during the current study are available from the corresponding author on reasonable request.

## Electronic supplementary material

Supplementary Figures
