# Supplementary material for: Sublingual house dust mite immunotherapy has no impact on decrease of circulating erythrocytes upon airway allergen challenge in allergic rhinitis
Source: Sci Rep. 2017 May 31;7:2555. doi: 10.1038/s41598-017-02321-y (PMC5451400; doi:10.1038/s41598-017-02321-y)
Supplement: Supplementary file 1 — Supplementary Figures [file 41598_2017_2321_MOESM1_ESM.pdf]

Sublingual house dust mite immunotherapy has no impact on decrease of circulating erythrocytes upon airway allergen challenge in allergic rhinitis

Galateja Jordakieva [1,2], MD; Michael Kundi [3], MD, PhD; Patrick Lemell, PhD [4]; René Zieglmayer, Dipl Ing [4]; Petra Zieglmayer [4], MD; Jasminka Godnic-Cvar [1], MD; Erika Jensen-Jarolim [2,5]*, MD

[1] Institute of Occupational Medicine, Department of Internal Medicine II, Medical University of Vienna, Vienna, Austria

[2] Institute of Pathophysiology and Allergy Research, Center of Pathophysiology, Infectiology and Immunology, Medical University of Vienna

[3] Center for Public Health, Medical University of Vienna

[4] Vienna Challenge Chamber (VCC), Vienna, Austria

[5] The Interuniversity Messerli Research Institute, Medical University Vienna, University of Veterinary Medicine Vienna, University of Vienna, Austria

***Corresponding Author**

erika.jensen-jarolim@meduniwien.ac.at

Prof. Dr. Erika Jensen-Jarolim, MD

Dept. of Pathophysiology and Allergy Research,

Center of Pathophysiology, Infectiology and Immunology,

Medical University Vienna

Währinger G. 18-20

1090 Vienna, Austria

Supplementary Figures

A)

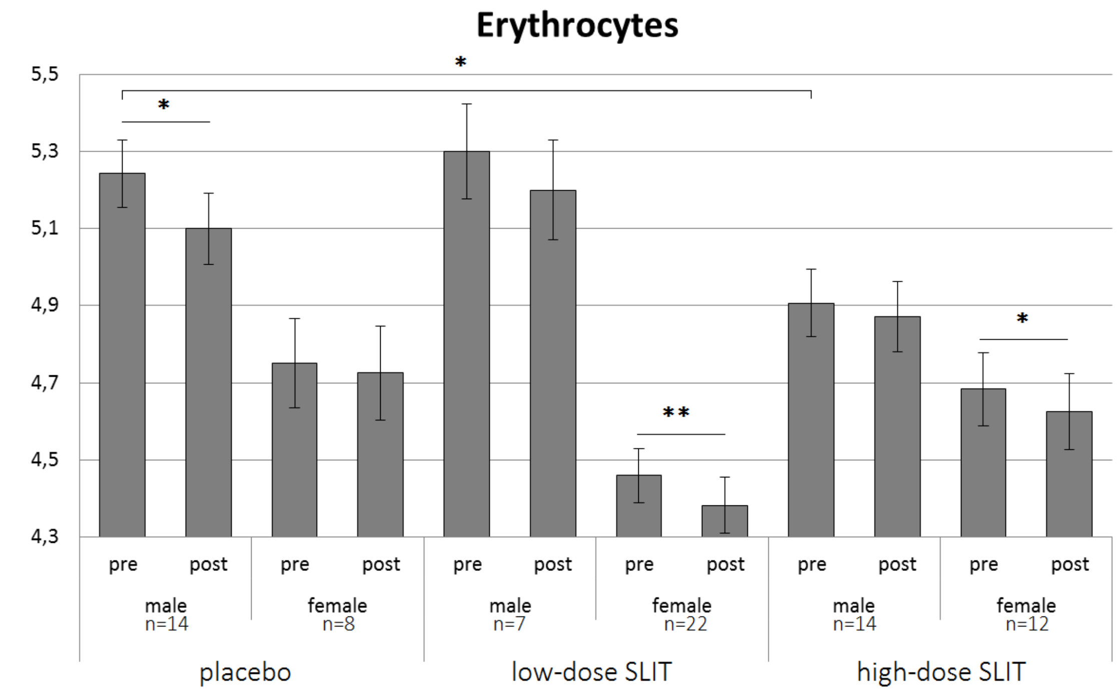

B)

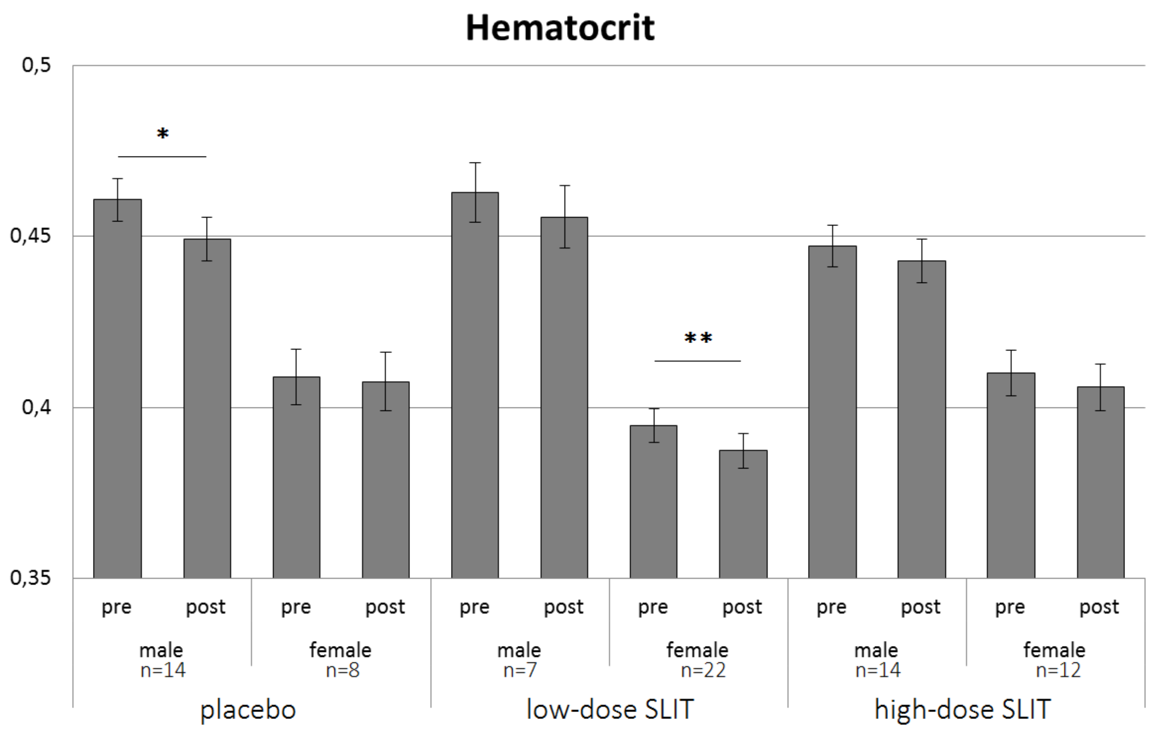

C)

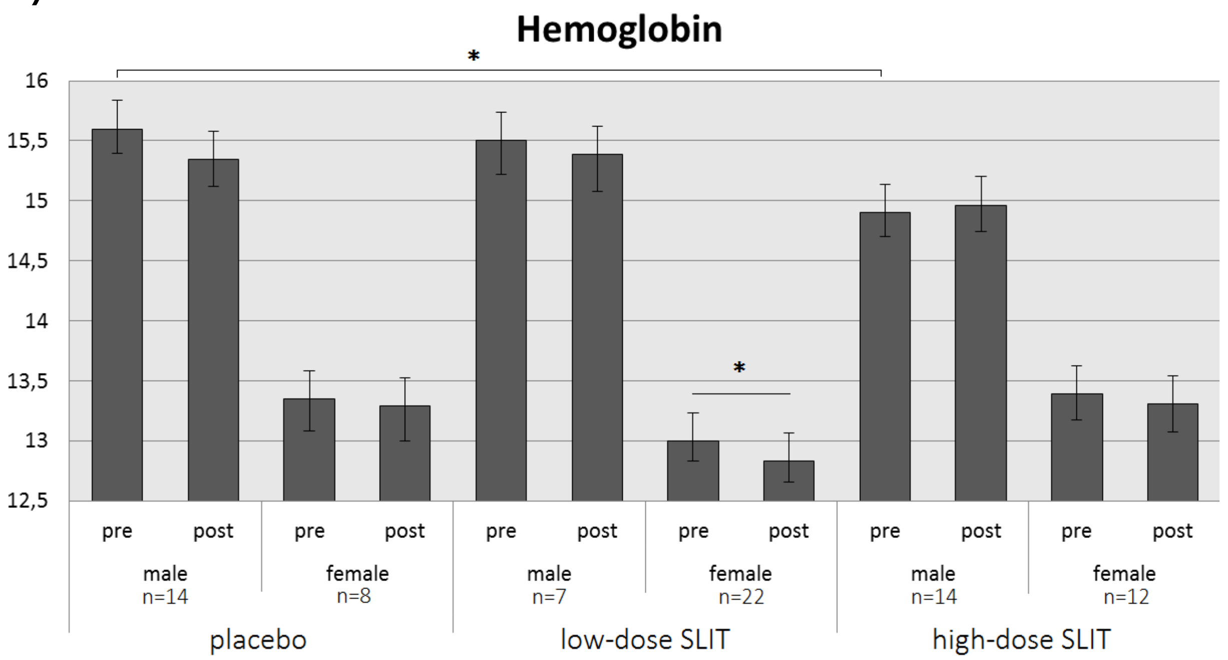

Supplementary Figure S1. Circulating erythrocytes numbers (A), hematocrit (B) and hemoglobin (C) levels before (pre) and after (post) airway allergen challenge with house dust mite extract in female and male subjects previously treated with placebo (group 1), low-dose (group 2) or high-dose (group 3) sublingual immunotherapy. Y-axis presents RBC counts x1012/l (A), Hct percentage (B) and Hg in g/dl (C), respectively. * p< .05; ** p< .01; *** p< .001;

A)

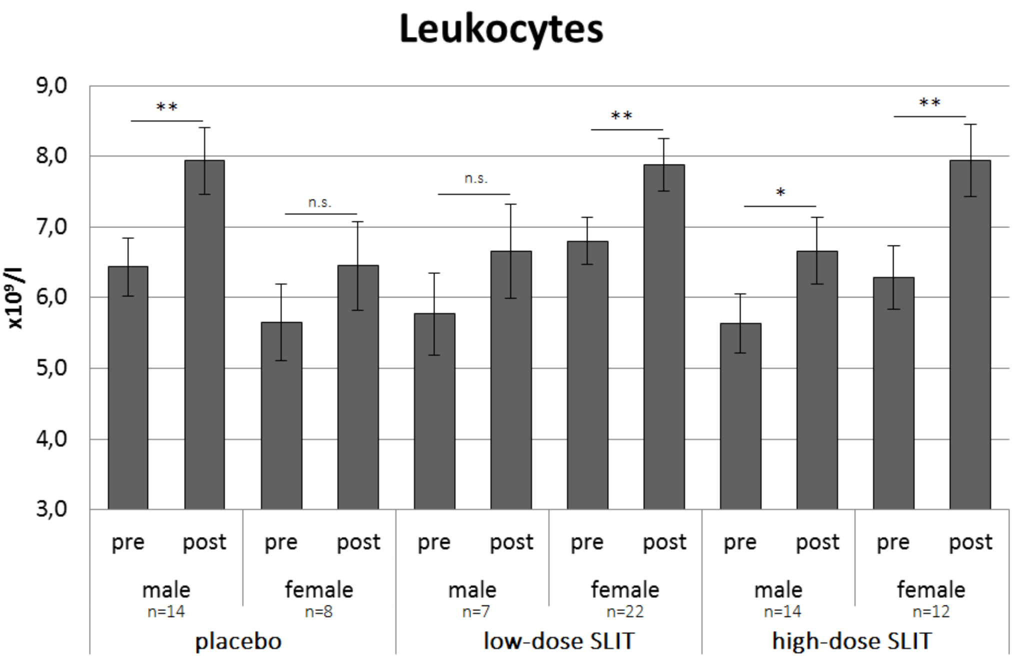

B)

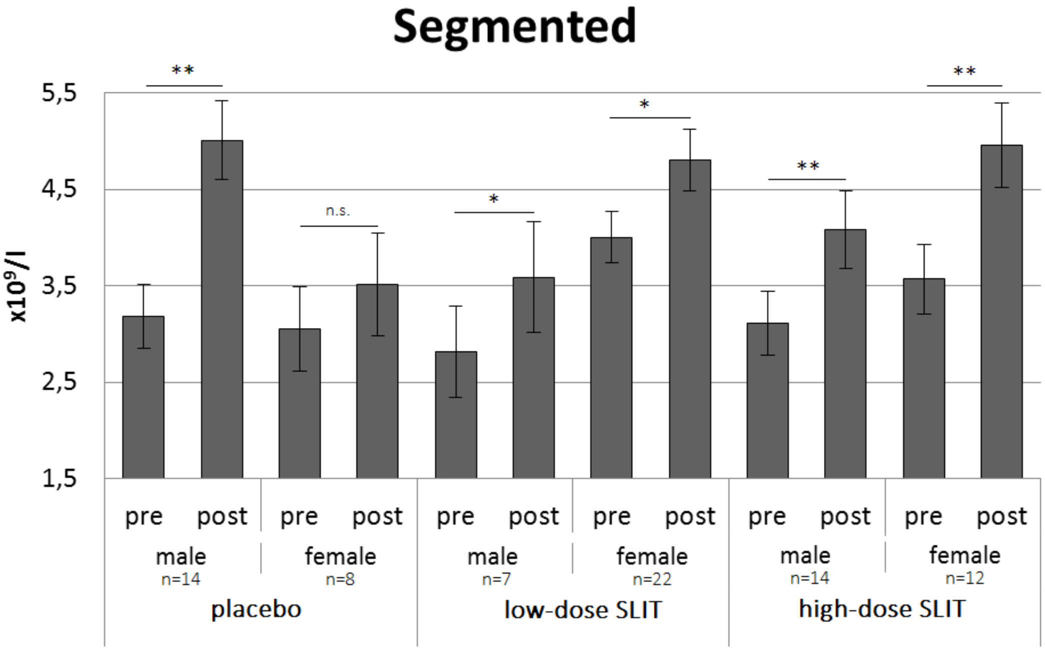

Supplementary Figure S2. Leukocytes (A) and segmented neutrophil numbers (B) before (pre) and after (post) airway allergen challenge with house dust mite extract in female and male subjects previously treated with placebo (group 1), low-dose (group 2) or high-dose (group 3) sublingual immunotherapy. Y-axis presents leukocytes (A) and segmented neutrophil counts (B) x 10^9/l, respectively. * p< .05; ** p< .01; *** p< .001; n.s. not significant;

A)

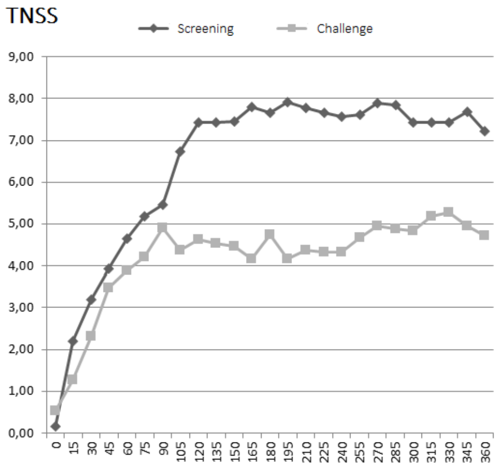

B)

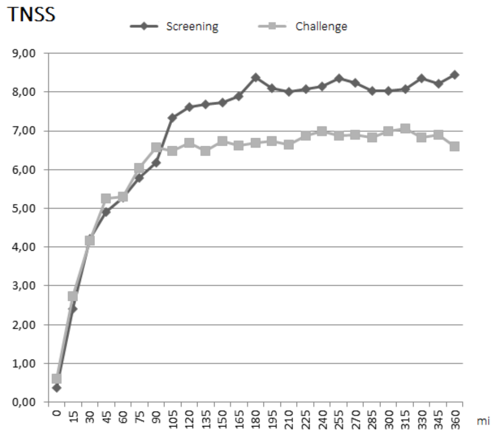

C)

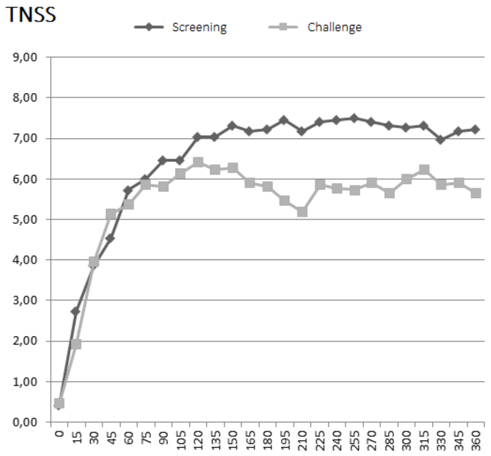

Supplementary Figure S3. Total nasal symptom scores (TNSS) before SLIT (Screening) and at time of study (Challenge) during airway allergen challenge with house dust mite extract all subjects treated with high-dose (A), low-dose (B) or placebo (C) sublingual immunotherapy. X-axis presents duration of allergen airway challenge in minutes; Y-axis presents respective symptom scores.
